# Respiration of permeabilized cardiomyocytes from mice: no sex differences, but substrate-dependent changes in the apparent ADP-affinity

**DOI:** 10.1038/s41598-019-48964-x

**Published:** 2019-08-29

**Authors:** Niina Karro, Martin Laasmaa, Marko Vendelin, Rikke Birkedal

**Affiliations:** Laboratory of Systems Biology, Department of Cybernetics, Tallinn University of Technology, Akadeemia tee 21, 12618 Tallinn, Estonia

**Keywords:** Energy metabolism, Cardiovascular biology

## Abstract

Sex differences in cardiac physiology are getting increased attention. This study assessed whether isolated, permeabilized cardiomyocytes from male and female C57BL/6 mice differ in terms of their respiration with multiple substrates and overall intracellular diffusion restriction estimated by the apparent ADP-affinity of respiration. Using respirometry, we recorded 1) the activities of respiratory complexes I, II and IV, 2) the respiration rate with substrates fuelling either complex I, II, or I + II, and 3) the apparent ADP-affinity with substrates fuelling complex I and I + II. The respiration rates were normalized to protein content and citrate synthase (CS) activity. We found no sex differences in CS activity (a marker of mitochondrial content) normalized to protein content or in any of the respiration measurements. This suggests that cardiomyocytes from male and female mice do not differ in terms of mitochondrial respiratory capacity and apparent ADP-affinity. Pyruvate modestly lowered the respiration rate, when added to succinate, glutamate and malate. This may be explained by intramitochondrial compartmentalization caused by the formation of supercomplexes and their association with specific dehydrogenases. To our knowledge, we show for the first time that the apparent ADP-affinity was substrate-dependent. This suggests that substrates may change or regulate intracellular barriers in cardiomyocytes.

## Introduction

In recent years there has been an increased focus on sex differences in cardiac physiology and pathology. Males and females differ not only genetically, but also in terms of their epigenetic control of gene regulation^1^. When it comes to cardiac contractility and susceptibility to cardiovascular diseases, rodent models exhibit sex differences in much the same way as humans^2^. It is recommended for future research that sex differences are taken into account^1^. This is particularly important in studies of transgenic mice, where underlying sex differences may lead to sex specific outcomes of genetic modifications. C57BL/6 mice are commonly used as background for transgenic mouse strains, and the present study started out as an assessment of whether the respiratory properties of healthy, adult C57BL/6 mouse cardiomyocytes differ between males and females.

Some previous studies addressing sex differences in cardiac metabolism have been performed. Overall, females have a higher capacity for fatty acid oxidation, whereas males have a higher glucose utilization^2^. But it is unclear whether mitochondrial regulation and respiration differ between sexes. A sex difference was found in adult humans, where the myocardial oxygen consumption *in vivo* is higher in women than in men^3^. One study found higher activities of complexes III and IV in young, male rats, but no difference in the respiration rates measured with different substrates^4^. Another study found no difference between young subjects, but old females have higher expression of some genes from complex I, III and IV^5^. A third study found that the activities of complexes I, II and IV do not differ between sexes in either young or old rats, but there is a transitional sex difference in the apparent ADP affinity as it declines with age^6^. The latter (the apparent ADP-affinity) estimates the overall diffusion restriction in cardiomyocytes. It relates, in part, to the extent of intracellular compartmentalization, which is believed to be crucial for the regulation of energy fluxes in cardiomyocytes^7–9^. With such variability, more studies are needed to see the whole picture.

The studies we have seen so far have assessed sex differences in respiration in the presence of either glutamate or pyruvate with malate, which activate respiration through complex I, or succinate, which activates respiration through complex II. Furthermore, the ADP-dependency is traditionally measured with only glutamate and malate as substrates^10^. However, under these conditions, the respiration rate is far from maximal^11^. Mitochondria *in vivo* use multiple substrates, and the flow of electrons from complexes I and II converge at the so-called Q-junction, where ubiquinone accepts electrons, that are transferred via cytochrome c to O_2_, resulting in the production of water. It is more physiological to assess the maximal respiration rates and apparent ADP-affinity with a combination of substrates, which leads to electron transfer through both complexes I and II and thus a higher respiration rate.

To perform this study, we used isolated, permeabilized cardiomyocytes. We could not use permeabilized fibres, which have been criticized for the assay of ADP-affinity, because the cells are in bundles and they may assemble on the top of the stirrer during the respirometry recordings. This may restrict the diffusion of ADP^12^. Indeed, oxygen diffusion is also restricted in permeabilized fibre bundles^13^. However, a more thorough dissection of the fibres may cause mechanical damage^14^. Therefore, we chose to measure on permeabilized cardiomyocytes from male and female mice the activities of respiratory complexes I, II and IV, and the maximal respiration rates through complex I, complex II and complex I + II. In addition, we recorded the ADP-dependency of respiration with only glutamate and malate (in order to compare with previous results) as well as with glutamate, malate, pyruvate, and succinate to fully activate respiration as in^11^.

## Results

### Animals and cell preparations

Cardiomyocytes were successfully isolated from 10 male and 10 female C57BL/6 mice. Their age, body weight and left tibial length are given in Table 1. The age of males and females was similar. Males had larger body weight (BW), tibial length (TL) and ratio of BW/TL than females (*p* < 0.001 in all three cases).

**Table 1:** Morphological parameters of 10 male and 10 female mice used in this study. Numbers are mean ± SEM. BW, body weight; TL, left tibial length; NS, not statistically significant.

	Age days	BW g	TL cm	BW/TL
Males	173 ± 10	33.5 ± 0.6	2.25 ± 0.01	14.9 ± 0.2
Females	169 ± 10	24.2 ± 0.5	2.16 ± 0.01	11.2 ± 0.2
*p*	NS	*p* < 0.001	*p* < 0.001	*p* < 0.001

*p* < 0.001, significant difference between males and females.

Characteristics of the cell suspensions are given in Table 2. The volume and viability of the cell suspensions did not differ between males and females. The cell suspensions from males tended to have a higher total number of isolated cardiomyocytes, but this difference was not significant due to the large variation. However, their protein content and activity of citrate synthase (CS) were significantly higher. When CS activity was normalized to protein content, there was no difference between males and females.

**Table 2:** Characteristics of the cell suspensions from 10 male and 10 female mice. Numbers are mean ± SEM. NS, not statistically significant.

	Viability %	Yield ml	Cell yield cells × 1000	Protein mg/ml	Citrate synthase μmol/min/ml	Citrate synthase μmol/min/g protein
Males	75.9 ± 1.7	0.384 ± 0.009	760 ± 53	17.87 ± 1.11	15.28 ± 0.93	864 ± 42
Females	77.9 ± 1.0	0.372 ± 0.009	653 ± 72	13.48 ± 1.17	10.53 ± 0.69	804 ± 41
*p*	NS	NS	NS	*p* < 0.05	*p* < 0.001	NS

*p* < 0.05 and *p* < 0.001, significant difference between males and females.

### Respiratory complexes I, II and IV

**Figure 1:**
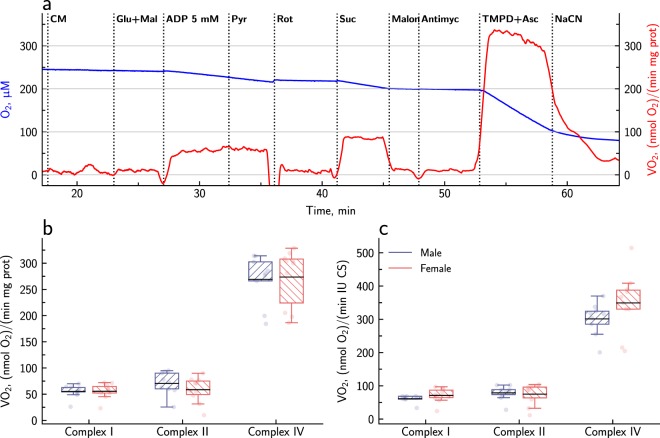
Assessment of the activities of the respiratory complexes I, II and IV in permeabilized cardiomyocytes from male and female mice. A representative trace of the oxygen consumption recorded during SUIT protocol 1 is shown in **a**, where the oxygen concentration in the chamber is shown in blue, and the oxygen consumption rate, i.e. the slope of the blue line, is shown in red. Panels (**b**,**c**) show the averaged data (9 males and 10 females) normalized to the protein content and CS activity, respectively. The individual datapoints are shown with pale dots, and the data are summarized with the box-and-whisker plots, where the black, solid line shows the mean, the box borders show the interquartile range from 25% to 75%, and the whiskers correspond to the Tukey box plot notation.

The activities of respiratory complexes I, II and IV were determined by sequential additions of their substrates and inhibitors. This is the SUIT 1 protocol described in the materials and methods. A representative example of such a recording is shown in Fig. 1a, and the average activities of complexes I, II and IV are shown in Fig. 1b,c. The maximal oxygen flux through complex IV stimulated by TMPD and ascorbic acid was much higher than when oxygen consumption was stimulated though complexes I and II. Irrespective of the normalization factor (proteins or CS), there was no statistically significant difference between males and females as determined by ANOVA and Bayesian ANOVA.

### Respiratory fluxes through complexes I and II

**Figure 2:**
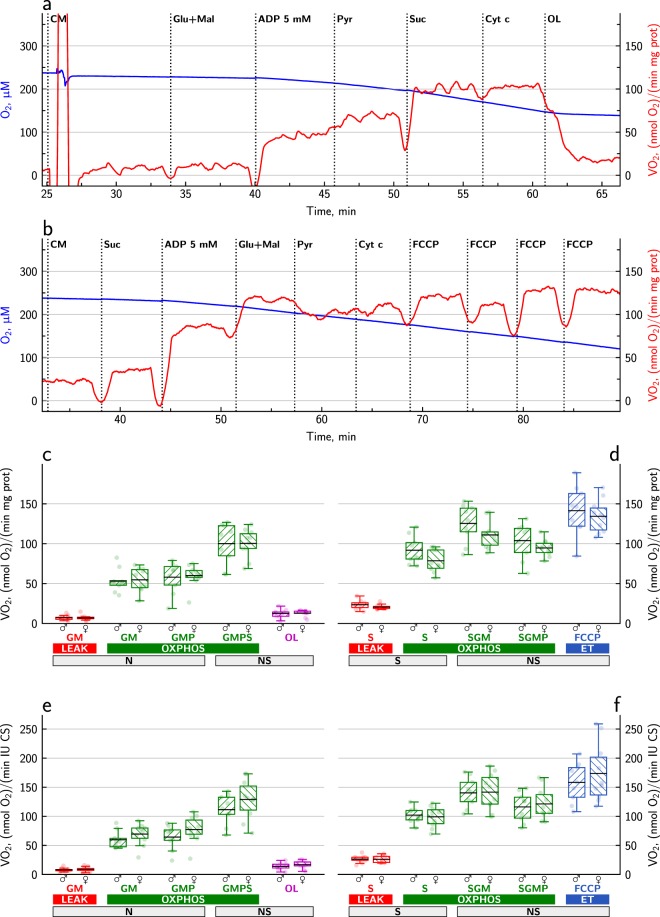
Assessment of respiration rate with multiple substrates activating both complexes I and II in permeabilized cardiomyocytes from male and female mice. Representative traces of oxygen consumption during SUIT protocol 2 and 3 are shown in (**a**,**b**), respectively. The blue line shows the oxygen concentration in the chamber, and the red line is the oxygen consumption rate, i.e. the slope of the blue line. Panels **c**–**f** show the averaged data from 9 males and 10 females. The individual datapoints are shown with pale dots, and the data are summarized with the box-and-whisker plots, where the black, solid line shows the mean, the box borders show the interquartile range from 25% to 75%, and the whiskers correspond to the Tukey box plot notation. In (**c**,**d**), the data from SUIT 2 and 3, respectively, are normalized to the protein content. In (**e**,**f**), the data from SUIT 2 and 3, respectively, are normalized to the CS activity.

In the presence of substrates for both complexes I and II, their flows of electrons converge at the Q-junction. With SUIT protocols 2 and 3 (see materials and methods), we wanted to assess whether the respiration rate of cardiomyocytes from males and females differ when complexes I and II are activated one after another to reach maximal respiration rate at the point where both complex I and II were fully activated. Representative traces are shown in Fig. 2a,b, respectively. Figure 2c–f show the average respiration rates in different states in males and females. In Fig. 2c,d, the rates are normalized to protein content, and in Fig. 2e,f, the rates are normalized to CS activity. The different normalizations did not change the overall picture, and there was no difference between males and females in any of the measurements. Therefore, the following points are valid for data from both males and females, normalized to protein or CS.

In SUIT protocol 2 (Fig. 2a,c,e), we first added cardiomyocytes (CM) and then activated complex I with glutamate and malate (Glu + Mal) to record leak_GM_ respiration. Permeabilization is normally carried out in the presence of substrates. This extra step was taken to verify that differences in leak respiration were indeed due to the addition of substrates. We then added 5 mM ADP to determine the respiration rate with glutamate and malate in the phosphorylating state (GM). The coupling efficiency with glutamate and malate was 0.882 ± 0.012 for females and 0.877 ± 0.013 for males. The addition of pyruvate (Pyr) to glutamate and malate sometimes caused a slight increase in the respiration rate, but not in all cases, and this was not significant (no difference between GM and GMP). Further activation of complex II with succinate (Suc) increased the respiration rate (GMPS) by 79 ± 17% in females and 85 ± 18% in males (GMP versus GMPS: *p* < 0.001, BF >100). Addition of cytochrome c (Cyt c), which is a quality test of the outer mitochondrial membrane intactness, increased respiration by 1.9 ± 0.7% in females and 0.7 ± 0.6% in males. This increase was significant according to a paired t-test (*p* < 0.05), but not according to the Bayesian paired t-test (BF <10). At the end of SUIT protocol 2, oligomycin (OL) was added to determine leak_OM_ in the presence of all substrates. Leak_OM_ was significantly higher than the leak with glutamate and malate (*p* < 0.001, BF >100).

In SUIT protocol 3 (Fig. 2b,d,f), we first added cardiomyocytes (CM) and then activated complex II with succinate (Suc) to record leak_S_ respiration. Leak_S_ respiration was significantly higher than leak_GM_ respiration (*p* < 0.001, BF >100). We then added 5 mM ADP to record the respiration rate with succinate in the phosphorylating state (S). In the absence of rotenone, succinate alone may lead to accumulation of oxaloacetate, which inhibits respiration^15^. However, a paired t-test showed that in the present experiments, there was no difference in respiration rate whether rotenone was present (as in SUIT 1) or absent (as in SUIT 3). The coupling efficiency with succinate was 0.747 ± 0.017 for females and 0.754 ± 0.017 for males. The additional activation of complex I by adding glutamate and malate (Glu + Mal) increased the respiration rate (SGM) by 44 ± 8% in females and 38 ± 8% in males (S versus SGM: *p* < 0.001, BF >100). But when pyruvate (Pyr) was added, the rate (SGMP) decreased by 14 ± 2% in females and 18 ± 2% in males (SGM versus SGMP: *p* < 0.001, BF >100). The SUIT protocols 2 and 3 converge at the point, where respiration is recorded with all four substrates, and there was no difference between GMPS and SGMP, which means that this rate does not depend on how you approach it. The addition of cytochrome c increased respiration by 6.7 ± 1.5% in females and 7.9 ± 2.1% in males. This increase was statistically significant (*p* < 0.001, BF >100). At the end of SUIT protocol 3, a titration with FCCP was performed to determine the capacity of the electron transfer system (ET). This allowed us to determine the oxidative phosphorylation control ratio (P/E), which was 83 ± 3% in females and 91 ± 6% in males for SGM and 71 ± 2% in females and 75 ± 4% in males for SGMP.

### ADP dependency of respiration

**Figure 3:**
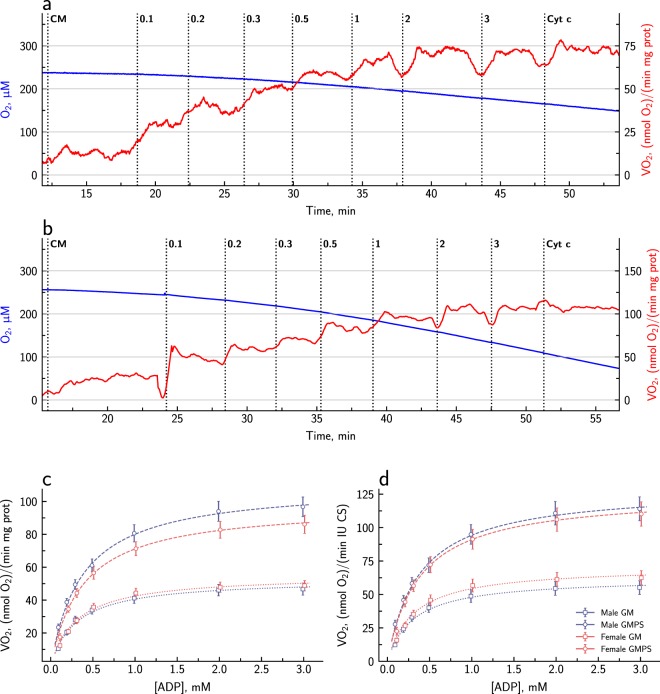
Assessment of the ADP-dependency of respiration in permeabilized cardiomyocytes from male and female mice. Representative traces of oxygen consumption during ADP_GM_- and ADP_SGMP_-titration are shown in (**a**,**b**), respectively. The blue line shows the oxygen concentration in the chamber, and the red line is the oxygen consumption rate, i.e. the slope of the blue line. Panels **c** and **d** show the averaged data (10 males and 10 females) normalized to the protein content and CS activity, respectively. The data points show mean ± SEM, and the lines show the hyperbolic curve obtained when the average V_max_ and K_M_ are inserted into the Michaelis-Menten equation.

The ADP-dependency of respiration has long been used as an indicator of the extent of intracellular diffusion restriction. To allow a comparison with data from other studies, we determined the relationship between respiration rate and ADP the traditional way with glutamate and malate as substrates (ADP_GM_-titration). We also determined this relationship with all substrates present and thus full activation of electron flow through complexes I and II (ADP_GMPS_-titration). Representative recordings are shown in Fig. 3a,b, respectively. The averaged data are shown in the plots in Fig. 3c,d, and various calculated values are given in Table 3.

**Table 3:** Characteristics of kinetic parameters determined by ADP-titration in 10 male and 10 female mice.

		GM		GMPS		GM vs GMPS	
		Male	Female	Male	Female	*p*	BF_10_
V_0_	nmol O_2_/min mg prot	4.9 ± 0.3	4.6 ± 0.4	15.8 ± 1.9	16.9 ± 1.4	≤0.001	≥100
	nmol O_2_/min IU CS	5.8 ± 0.5	5.9 ± 0.7	18.0 ± 1.8	21.6 ± 2.1	≤0.001	≥100
V_max_	nmol O_2_/min mg prot	53 ± 4	55 ± 4	110 ± 7	98 ± 6	≤0.001	≥100
	nmol O_2_/min IU CS	62 ± 6	71 ± 6	130 ± 10	125 ± 10	≤0.001	≥100
K_M_	μM	302 ± 3	305 ± 2	374 ± 3	376 ± 1	≤0.001	≥100
ACR		5.2–14.1	8.1–13.5	4.1–12.7	3.8–8.0		
CE		0.912 ± 0.007	0.922 ± 0.003	0.880 ± 0.011	0.861 ± 0.007	≤0.001	≥100

Numbers are mean ± SEM. There was no difference between males and females. The two protocols, ADP_GM_- and ADP_GMPS_-titration, were compared with ANOVA (*p*) and Bayesian ANOVA (BF_10_).

In these experiments, there were no differences between males and females. However, there was a significant difference between the two protocols, ADP_GM_-titration and ADP_GMPS_-titration (Table 3). Both leak and maximal respiration rate were higher in the ADP_GMPS_-titration. As a result, the coupling efficiency was significantly higher in the ADP_GM_-titration than in the ADP_GMPS_-titration. Furthermore, the apparent K_M ADP_ was ~25% higher in the ADP_GMPS_-titration than in the ADP_GM_-titration.

### Variation of the data

The variation of the data is described by the coefficient of variation (CV), which is generally defined as the ratio of the standard deviation to the mean. The CV is an indicator of the quality of the overall dataset. We provide the CV for pooled data from males and females in Table 4. The CV was lower for factors, which were not normalized the protein content or CS activity, such as the coupling efficiency and the apparent K_M ADP_.

**Table 4:** The coefficient of variation (CV; defined as the ratio of standard deviation to the mean) for data from male and female mice together.

Protocol	Value	CV, %
Protein measurements		26.6
CS activity		27.2
SUIT 1	Complex I	23.8
	Complex II	36.3
	Complex IV	18.4
SUIT 2	GM	26.4
	GMPS	21.3
	CE_GM_	4.3
SUIT 3	S	19.8
	SGMP	17.9
	CE_S_	6.8
ADP_GM_-titration	V_max_	21.9
	K_M_	2.9
	CE_GM_	2.2
ADP_GMPS_-titration	V_max_	20.3
	K_M_	1.7
	CE_S_	4.2

## Discussion

Our main finding is that the respiration of permeabilized cardiomyocytes from healthy C57BL/6 mice in the presence of substrates that activate both complexes I and II did not differ between males and females. Our results are in agreement with and complement previous studies on rat hearts, where the separate fluxes through complexes I and II were assessed^4,6^. We found that the activity of CS, which is a validated marker of mitochondrial content^16^, was similar in males and females when normalized to the protein content of the cell suspension (Table 2). Furthermore, the ADP-dependency of respiration was also similar in males and females (Fig. 3 and Table 3).

It is important to note the limitations of the present experiments, where the cardiomyocytes were isolated and thus taken out of their physiological context. They were permeabilized so that the surrounding solution controlled their intracellular environment, and the recordings were performed at 25 °C, because this is the temperature at which several studies on intracellular compartmentalization and its mechanistic aspects were done. For example, experiments performed at 25 °C demonstrated the strong coupling between ATPases and mitochondrial respiration^7,17,18^, found that the intracellular diffusion restrictions are localized in certain areas of the cardiomyocytes^19,20^, estimated diffusion coefficients and their anisotropy in cardiomyocytes^20,21^, and estimated the number of open voltage-dependent anion channels (VDACs) in cardiomyocyte mitochondria^22^ based on the known VDAC permeability under these conditions^23^. In this context, when interpreting the data, we have to take into account the temperature at which our results are applicable and acknowledge that we have not determined sex differences in Q_10_ of key metabolic enzymes, substrate utilization and regulation of mitochondrial function^11^. Furthermore, sex differences in the action of saponin due to differences in membrane lipid composition cannot be excluded. In any case, our results cannot be directly extrapolated to the situation *in vivo*, where sex differences in, for example, vascularization^24^, blood pressure and its regulation^3,25,26^, substrate utilization^3,5^, electrophysiology, and mechanical performance^27^ can affect oxygen availability and consumption of the heart. Our study does, however, show that in the experimental setting of the current study there are no differences between males and females in terms of CS activity, respiratory capacity, and apparent ADP-affinity.

Our data illustrate well how the coupling efficiency of respiration varies with the substrates. When comparing the ADP_GM_-titration and ADP_GMPS_-titration, the coupling efficiency with GM was slightly, but significantly higher than that with GMPS (Table 3). Taken together with the data from SUIT protocol 2, the coupling efficiency in the presence of glutamate and malate was around 0.9. In comparison, the coupling efficiency with succinate was around 0.75 and thus much lower. Indeed, leak respiration depends on the membrane potential in a manner that is inversely related to the H^+^/O_2_ stochiometry^28^. Assuming, as in^28^, that complex I substrates translocate 10 H^+^/O_2_ and complex II substrates translocate 6 H^+^/O_2_, then for a given proton flux, the O_2_ consumption is higher with succinate (0.17 O_2_/H^+^) than with glutamate, malate, and pyruvate (0.10 O_2_/H^+^). As the capacities of complexes I and II were similar (Fig. 1), the higher leak with complex II substrates led to a lower coupling efficiency.

Pyruvate had little or no additional stimulatory effect on respiration when added to glutamate and malate (Fig. 2c,e). This suggests that complex I was already fully activated by glutamate and malate. Our results are in contrast to a study on permeabilized mouse heart fibres, where the addition of pyruvate to glutamate and malate caused nearly a doubling of the respiration rate through complex I^11^. On the other hand, a study on horse skeletal muscle mentioned a transient lowering of respiration rate, when pyruvate was added to glutamate and malate^29^. We have no explanation for this discrepancy between studies. In contrast, when pyruvate was added to succinate, glutamate and malate, it caused a 16% decrease in the respiration rate. This effect was consistently observed on the raw traces (Fig. 2b,d,f), and it was statistically highly significant. As most studies record respiration with succinate either in the presence of rotenone or after addition of complex I substrates, we have not been able to find any mention of this in the literature. In rabbit liver mitochondria the addition of 20 mM pyruvate did not inhibit succinate oxidation^30^. We note that the experiments in^30^ were performed in the presence of rotenone, which inhibits complex I, which suggests that pyruvate does not inhibit succinate uptake and that its inhibitory effect in the current study is through complex I. Complex I binds to a number of NAD-linked dehydrogenases in the mitochondrial matrix such as pyruvate dehydrogenase, but not malate or glutamate dehydrogenase^31^. Furthermore, supercomplexes are formed between respiratory complexes I, III and IV, whereas the involvement of complex II in supercomplexes is controversial^32–34^. The structural association of dehydrogenases to complex I, and thus to complexes III and IV, causes the formation of a so-called „metabolome“, where the electrons pass straight from the dehydrogenase and through the electron transport chain^34^. It is conceivable that the addition of pyruvate initiates such a „channeling of electrons“. Pyruvate lowers the respiration rate, if the phosphorylation system limits the proton flux. As noted above, the electron transfer chain consumes 0.10 O_2_/H^+^ for complex I substrates and 0.17 O_2_/H^+^ for complex II substrates. If the phosphorylation system is already at its maximum and cannot use protons at a faster rate, a shift towards more use of complex I substrates will lower the respiration rate.

The recording of respiration with succinate alone may cause accumulation of oxaloacetate, which is a potent inhibitor of complex II^15^. This could be an issue in SUIT protocol 3, which was performed to assess how activation of complex I after complex II affects respiration rate. Indeed, isolated rat heart mitochondria display a three-phasic respiratory response to stimulation of respiration with ADP and succinate^35^. However, we saw no evidence of such inhibition in the present experiments. First, the respiration rate was stable during the recordings (Fig. 2b). Second, a paired t-test showed no difference between respiration rate recorded in the presence (SUIT 1) and absence (SUIT 3) of rotenone. Third, the respiration rate in the presence of all substrates was the same whether the recording had started with succinate alone (SGMP) or glutamate and malate (GMPS; Fig. 2). Our results are in agreement with that the inhibition by oxaloacetate depends on the ratio of succinate to oxaloacetate so that excess succinate relieves the inhibition and leads to full activation of Complex II^36,37^. It is also possible that ATP, which is an effective activator of complex II^38^, plays a role. Although endogenous ATP does not seem to be important in isolated mitochondria^37^, mitochondria in permeabilized cardiomyocytes are preserved in their structural environment. The resulting preservation of macro- and microcompartments may cause a locally high ratios of ATP/ADP, which regulate the activity of complex II.

To our knowledge, we show for the first time that the apparent ADP-affinity of respiration is substrate-dependent. The K_M ADP_ was modestly higher (25%) in the presence of GMPS compared to GM, but the difference was highly significant (Table 3). In the kinetics of pure enzymes, the K_M_ is independent of V_max_. But the situation in permeabilized cardiomyocytes is not straightforward, because it depends not only on enzyme kinetics but also on the rate of diffusion from the external medium to the adenine nucleotide translocase (ANT) in the inner membrane of all mitochondria. The diffusion is affected by hitherto unidentified barriers inside the cells^39,40^, and only ~2% of VDACs in the outer mitochondrial membrane is accessible to external ADP^22^. Removal of the myofilaments as in so-called „ghost fibres“ does not affect the ADP-affinity^41^. The most likely candidates to form these barriers are membrane structures such as the sarcoplasmic reticulum and the outer mitochondrial membrane. A study on lobster giant muscle fibres concluded that muscles from mammals and fish have a common intracellular diffusion barrier, which could very well be the sarcoplasmic reticulum^42^. A 3D model of rat cardiomyocytes supported this hypothesis^20,43^, and it is consistent with the close contact between mitochondria and the sarcoplasmic reticulum^44^. The outer mitochondrial membrane may also form a barrier and point of regulation because the permeability of VDAC is regulated by tubulin^45^. It is important to note that these theories do not exclude each other as shown in the estimation of the relative contribution of diffusion barriers^22^. Our finding that the K_M ADP_ varies with the substrates suggests that some of the diffusion barriers change in a substrate-dependent manner. It opens up for more studies addressing how the permeability of the outer mitochondrial membrane is affected, i.e. which barriers change the accessibility of VDAC to ADP, and whether this change relates to substrate uptake and/or respiration rate.

Whereas the apparent K_M ADP_ varied with the substrates, it did not differ between males and females (Table 3). These results must be interpreted with caution, because the apparent ADP-affinity only estimates the overall diffusion restriction between the solution surrounding the permeabilized cardiomyocytes and ANT in the mitochondrial inner membrane. As multiple intracellular structures may reduce the access of cytosolic ADP to mitochondria^9^, the K_M ADP_ alone is insufficient to establish whether it is due to a reduction of mitochondrial outer membrane permeability or reduction of diffusion^46^. Other experiments are required to estimate the partitioning of intracellular diffusion restrictions between different intracellular structures: following ATPase coupling with respiration^47,48^, temporal characterization of mitochondrial response to stimulation^19^, or heterogeneity of intracellular mitochondrial response^22^. In this work, we have shown that the apparent ADP-affinity is the same for cardiomyocytes isolated from male and female mice. It does not exclude that the diffusion restriction in males and females is induced by different intracellular structures. But if there are differences between male and female mouse cardiomyocytes, the differences balance each other out resulting in similar apparent ADP-affinities.

The overall quality of the present dataset was assessed by the CV of the data, the coupling efficiency of the respiration, and the increase in respiration rate induced by cytochrome c addition. The respiration rates had a CV similar to that of permeabilized fibres^49^, whereas the coupling efficiencies and K_M ADP_ had lower CVs (Table 4). The fact that the non-normalized parameters (coupling efficiency and K_M ADP_) had a smaller variability suggests that a large part of the variation was due to variation in the normalization factors and not in the preparation itself. Indeed, protein content and CS activity had CVs similar to those of the respiration rates. The coupling efficiency is defined as (P-L)/P, where P is the respiration rate at saturating concentrations of ADP, P_i_, and substrates (corresponding to state 3), and L is the leak respiration occurring in the absence of ATP synthesis and determined either before addition of ADP or after addition of oligomycin to inhibit the F_1_/F_0_-ATPase^15^. It is replacing the respiratory acceptor control ratio, ACR or RCR, which is calculated as State 3/ State 4 respiratory rate^50^. The coupling efficiency must be interpreted with caution because, as shown in the present study, it varies with the substrates. Lemieux *et al*. also found a higher coupling efficiency for PM than for GM (0.82 and 0.69 at 25 °C, respectively)^11^. In comparison, the coupling efficiencies with complex I and I + II substrates in the present study were even higher. The addition of cytochrome c increased respiration rate only in some cases and not by more than 8%. This increase was smaller than for permeabilized fibres^11^. Taken together, this suggests that the preparation in the present study was sound.

In summary, we have demonstrated that permeabilized cardiomyocytes from male and female mice do not differ in terms of CS activity, respiratory capacity, and apparent ADP-affinity. The lowering of respiration rate, when pyruvate was added to succinate, glutamate and malate, may be explained by intramitochondrial compartmentalization induced by the formation of supercomplexes and their association with specific dehydrogenases. Lastly, our finding that the apparent ADP-affinity was substrate-dependent suggests that substrates may change or regulate intracellular barriers in cardiomyocytes.

## Methods

All animal procedures were carried out according to directive 2010/63/EU of the European Parliament and had been approved by the Project Authorisation Committee for Animal Experiments in the Estonian Ministry of Rural Affairs.

### Animals

C57BL/6J Ola Hsd mice were obtained from Envigo RMS B.V. (The Netherlands) at the age of 6–8 weeks. They were kept for several weeks at our local animal facility with free access to water and food (V1534-000 Rat/mouse maintenance from Ssniff Spezialdiäten GmbH, Germany), an ambient temperature of 22–22.8 °C, and a 12:12 hours light:dark cycle.

### Isolation of cardiomyocytes

Cardiomyocytes were isolated using a slightly modified version of a method described previously^51^. The mice were anesthetized with a mixture of ketamine/dexmedetomidine (150 mg/kg and 0.5 mg/kg, respectively) and received an injection of 250 U of heparin to prevent blood coagulation. When the toe-pinch reflex was absent, the animal was killed be cervical dislocation. The heart was excised and immediately placed in ice-cold wash solution. It was cannulated via the aorta on a Langendorff perfusion system thermostatted to ~37 °C. The heart was first perfused with wash solution at a constant pressure of 80 cm H_2_O. When the heart was washed free of blood, the perfusion was switched to a constant flow with digestion solution containing 0.25 mg/ml Liberase DL (Roche) and 1.36 mg/ml of dispase II (Roche). After 10–15 minutes, the heart was soft, and the perfusion was stopped. The ventricles were cut into smaller pieces, transferred to a beaker with digestion solution and incubated further at 37 °C with gentle shaking until the tissue started falling apart. Cells were harvested with a pasteur pipette several times and filtered through a 100 µm cell strainer (EASYstrainerTM Cell Strainer, Greiner Bio-One) into a vial with sedimentation solution. The viable cells were separated by sedimentation or by centrifugation for 2 min at 300 rpm/12 g in an Eppendorf 5810 R centrifuge with an F-34-6-38 rotor (Eppendorf AG.). During the first washes, extracellular Ca^2+^ was gradually increased to 2 mM to ensure Ca^2+^ tolerance of the cells. Then, extracellular Ca^2+^ was washed out again by washing the cells three times with 5 ml of sedimentation solution. The isolated cells were stored in this solution at room temperature until use.

To assess the quality of the cell preparation, the yield of the cell suspension was measured with a 1000 μl pipette (Eppendorf), and a 1:10 dilution of the cells were counted in a chamber to estimate the total number of cells as well as the viability (the percentage of live cells relative to the total number of cells).

### Respiration measurements

Measurements of oxygen consumption were carried out using a Strathkelvin RC 650 Respirometer equipped with six 1302 O_2_-electrodes connected via a 929 Oxygen System interface (all from Strathkelvin Instruments Limited, UK) to a computer. The respirometer was thermostatted to 25 °C (Julabo F12-ED, JULABO Labortechnik GmbH), and each chamber contained 2 ml respiration solution (see composition below). The rate of oxygen consumption [nmol O_2_/(min mg protein)] was calculated by our home-made software, IOCBioStrathKelvin. The latter is open source software and freely available at https://github.com/iocbio/iocbio.

In the substrate-inhibitor titration protocol 1 (SUIT 1) to determine the activity of the respiratory complexes I, II and IV the following final concentrations were added sequentially: 4 μl of cell suspension (CM), 5 mM glutamate and 2 mM malate (Glu + Mal), 5 mM ADP, 5 mM pyruvate (Pyr*)*, 0.5 μM rotenone (Rot), 15 mM succinate (Suc), 5 mM malonic acid (Malon), 2.5 μM antimycin A (Antimyc), 10 mM ascorbate (Asc) and 3 mM *N*,*N*,*N*′,*N*′-tetramethyl-*p*-phenylenediamine (TMPD), and 5 mM sodium cyanide (NaCN).

In the SUIT 2 protocol, the following final concentrations were added sequentially: 5–10 μl of cell suspension, 2 mM malate (M), 5 mM glutamate (G), 5 mM ADP (no. J60672, Alfa Aesar), 5 mM pyruvate (P), 15 mM succinate (S), 10 μM cytochrome *c* (Cyt c), 5 μM oligomycin (OL; no. 579-13-5, Cayman Chemical).

In the SUIT 3 protocol, the addition of subtrates was inversed: 5–10 μl of cell suspension, 15 mM succinate (S), 5 mM ADP, 2 mM malate (M), 5 mM glutamate (G), 5 mM pyruvate (P), 10 μM cytochrome *c* (Cyt c). Uncoupling was performed by stepwise titration of carbonyl cyanide *4-*trifluoromethoxyphenylhydrazone (FCCP) in 5 μM steps until a maximum was reached.

For recordings of ADP kinetics, 5–10 μl of cell suspension was added to the respirometer chambers. The ADP concentration was increased in steps, and respiration rate was allowed to reach steady state before the addition of more ADP. ADP-titrations were carried out in the presence of 5 mM glutamate and 2 mM malate only (ADP_GM_-titration) and in the presence of 5 mM glutamate, 2 mM malate, 5 mM pyruvate and 15 mM succinate (ADP_GMPS_-titration). For most experiments, 10 μM cytochrome *c* (Cyt c) was added at the end of the ADP-titration to test the intactness of the outer mitochondrial membrane.

### Normalization

To measure cell suspension protein content, 50 μl of cell suspension and 50 μl of sedimentation solution were incubated with 2.5 μl 30% sodium dodecyl sulfate (SDS) solution at 80 °C for 30 min. The samples were frozen until determination of their protein content in a BioSpec-nano spectrophotometer (Shimadzu Scientific Instruments Inc., Maryland, USA), which recorded the absorption spectra from 220–800 nm. The protein content of a 1:10 dilution of each sample was measured 3–6 times. The absorption at 280 nm was background corrected by subtracting the absorption at 330 nm, and protein content was calculated using the molecular weight and extinction coefficient of BSA (66 400 g mol^−1^ and 43 824 M^−1^ cm^−1^, respectively). The protein content of each cell suspension was corrected for the protein content of the corresponding sedimentation solution.

To measure CS activity, 50 μl of cell suspension was frozen at −80 °C until use. CS activity was determined spectrophotometrically in an Evolution 600 spectrophotometer (Thermo Fisher Scientific) equipped with a Peltier water-cooled cell changer (SPE 8 W, Thermo Fisher Scientific) to maintain temperature at 25 °C. At the day of measurements, the cell suspension was diluted 1:10 in 100 mM Tris buffer containing 0.5% Triton X-100 (pH 8.1) and incubated for 4 h at 4 °C. The assay system contained in a total volume of 1 ml: 100 mM Tris buffer (pH 8.1), 0.1 mM 5,5-dithiobis(2-nitrobenzoate) (DTNB), 0.3 mM acetyl-CoA, and 10 μl of the diluted (1:10) cell suspension. The change in absorbance was recorded at 412 nm before (for reference) and after addition of 0.5 mM oxaloacetate for 3 min. All measurements were performed in triplicate and the results averaged. The enzyme activity was calculated using the extinction coefficient for thionitrobenzoate (TNB), which is 14150 M^−1^ cm^−1^ at 25 °C^52^.

### Solutions

The wash solution consisted of (in mM) 117 NaCl (no. 71379, Sigma-Aldrich), 5.7 KCl (no. P-5405, Sigma-Aldrich), 1.5 KH_2_PO _4_ (no. P-0662, Sigma-Aldrich), 4.4 NaHCO_3_ (no. S-6014, Sigma-Aldrich), 1.7 MgCl_2_ (no. 63068, Sigma-Aldrich), 21 HEPES (no. H-3375, Sigma-Aldrich), 20 taurine (no. 86329, Sigma-Aldrich), 11.7 glucose (no. 158968, Sigma-Aldrich) and 10 2,3-butanedione monoxime (no. B0753, Sigma-Aldrich). pH was adjusted to 7.4 with NaOH.

For the digestion solution, 0.25 mg/ml Liberase DL (no. RD05466202001, Roche) and 1.36 mg/ml Dispase II (no. 04942078001, Roche) was added to 20 ml of the wash solution.

For the sedimentation solution, 2 mM pyruvate (no. P-2256, Sigma-Aldrich), 10 µM leupeptin (no. 11034626001, Roche), 2 µM soybean trypsin inhibitor (no. 93619, Sigma-Aldrich), and 3 mg/ml BSA (no. 10775835001, Roche) were added to 40 ml of the wash solution.

The respiration solution contained (in mM) 110 sucrose (no. S-1888, Sigma-Aldrich), 60 K-lactobionic acid (no. L-2398, Sigma-Aldrich), 3 KH_2_PO_4_ (no. P-0662, Sigma-Aldrich), 3 MgCl_2_ (no. 63068, Sigma-Aldrich), 20 HEPES (no. H-3375, Sigma-Aldrich), 20 taurine (no. 86329, Sigma-Aldrich), 0.5 EGTA (no. 71379, Sigma-Aldrich), 0.5 DTT (no. D-0632, Sigma-Aldrich). pH was adjusted to 7.1 with KOH. Immediately before use, 5 mg/ml BSA (no. 10775835001, Roche) and 25 μg/ml saponin (no. 47036, Sigma-Aldrich) were added. In contrast to digitonin^53^, saponin is effective across a range of tissue concentrations^49^. The choice of saponin concentration was determined in preliminary experiments (not shown) showing that this concentration led to permeabilization within a couple of minutes. Increasing the concentration to 50 μg/ml, as often used for the permeabilization of fibres, lowered the respiration rate. A concentration of 25 μg/ml was also shown in another study to be optimal for permeabilized cells^54^.

Stock solutions were prepared according to the guidelines in^15^.

### Statistics

Unless stated otherwise, the values are shown and mean ± standard error of the mean. Statistical analyses were performed using the free software JASP. SQL and Python scripts were written to fetch the data from our database into a file format suitable for JASP input. The characteristics of mouse morphology and cell suspensions were analyzed by a Student’s t-test. For the respiration experiments, the effects of condition and sex were analyzed by a two-way repeated measures ANOVA as well as a Bayesian two-way repeated measures ANOVA. The results of both are included. *p* < 0.05 was considered statistically significant. For interpretation of the Bayes Factor (BF), we used the categories as in^55^, where BF <10 was not significant, 10≤ BF >30 was strong evidence, 30≤ BF >100 was very strong evidence, and BF ≥100 was extremely strong evidence for the tested hypothesis.

The datasets generated during the current study are available from the corresponding author on reasonable request.
